# Supplementary material for: Fecal Microbiome Composition Correlates with Pathologic Complete Response in Patients with Operable Esophageal Cancer Treated with Combined Chemoradiotherapy and Immunotherapy
Source: Cancers (Basel). 2024 Oct 29;16(21):3644. doi: 10.3390/cancers16213644 (PMC11545537; doi:10.3390/cancers16213644)
Supplement: Supplementary file 1 [file cancers-16-03644-s001.zip › cancers-3239192-Figure S1.pdf]

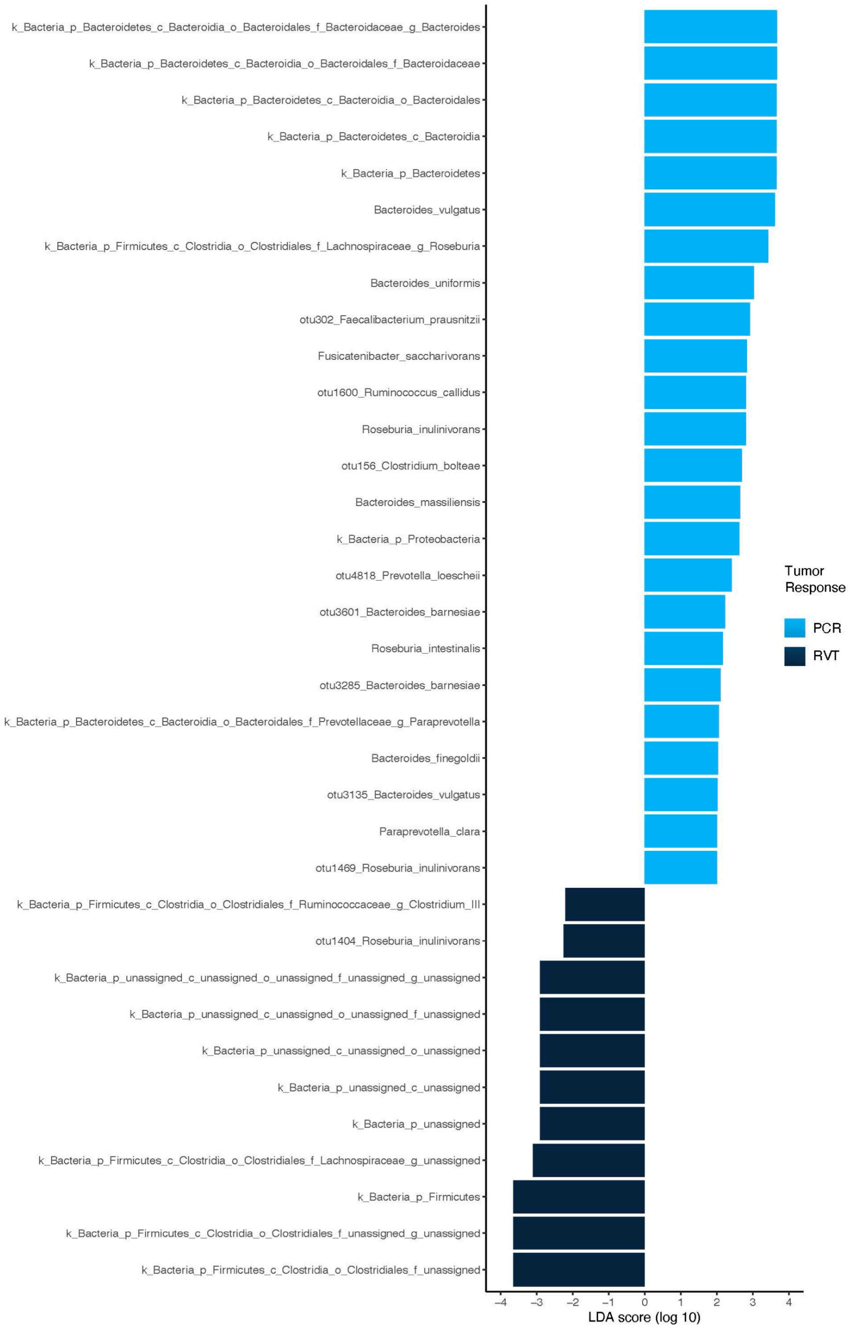

Figure S1. Significant taxonomic features by response group. Significant taxonomic features that are mostly likely to explain differences between tumor response groups using linear discriminant analysis effect size (LEfSe). The direction of the bar and the color indicate enrichment in tumor response group.
